# Is Early Conversion to mTOR Inhibitors Represent a Suitable Choice in Renal Transplant Recipients? A Systemic Review of Medium-term Outcomes

**Published:** 2017-05-01

**Authors:** J. Kumar, I. Reccia, T. Kusano

**Affiliations:** Department of Surgery and Cancer, Imperial College London, London, UK

**Keywords:** Adverse events, Calcineurin inhibitors, Graft failure, Kidney transplantation, mTOR inhibitors

## Abstract

**Background:**

Immunosuppressive therapies are important parts of renal transplantation.

**Objective:**

To assess the present literature on the effectiveness of early introduction of mTOR inhibitors with or without calcineurin inhibitors (CNI) in renal transplant recipients in terms of renal functioning and graft survival.

**Methods:**

The current literature was reviewed following PROSPERO approval, assessing the role of immunosuppressive agent, mTOR inhibitors as an alternative to CNI within 6 months of renal transplantation by searching PubMed, EMBASE, Cochrane, Crossref, and Scopus.

**Results:**

6 articles of early withdrawal of CNI and introduction of mTOR inhibitors within 6 months of renal transplantation were sought. Glomerular filtration rate (GFR) and serum creatinine were better in mTOR inhibitor group at 12 months. Biopsy-proven acute rejection (BPAR) was significantly higher in mTOR inhibitor group, though survival was comparable.

**Conclusion:**

On the basis of present literature, the early introduction of mTOR inhibitors causes substantial CNI minimization. The mTOR inhibitors are more favorable due to their complementary mechanism of action and favorable nephrotoxicity profile, better glomerular filtration, and lower serum creatinine with equivalent survival. However, the higher rejection rate may influence the use of these regimens in patients with moderate to high immunological risk.

## INTRODUCTION

Over time, the advancement in the immunosuppressive therapies has brought real success for renal transplant patients [1]. The calcineurin inhibitors (CNIs), cyclosporine A (CsA), and tacrolimus (Tac) were put in motion in 1980s and since then it is part of well-founded immunosuppressive regimen with more than 90% one-year graft survival while maintaining a rejection rate of less than 20% [2]. However, the excellent results of short-term allograft survival have not lasted long enough due to slow, steady decline in renal functioning in terms of GFR below 50% in a period of 10 years [3]. Review of literature suggested chronic allograft nephropathy (CAN) as the major cause of late graft loss in 40% of kidney transplant recipients, with 43% mortality due to delayed functioning graft (DFG). The cardiovascular diseases and malignancies are considered major causative attributes of DFG [4]. 

The CNI-induced nephrotoxicity is labeled as a foremost determining factor of long-term graft failure in 96.8% of allograft biopsies. The increased production of vasoconstrictors, thromboxane and endothelin, with decreasing level of vasodilators, such as nitric oxide, prostaglandin E2, and prostacyclin are considered important determinants [5, 6]. Nankivell, *et al*, demonstrated attestation of chronic CNI toxicity in more than 50% of kidney allograft biopsies following 10 years of transplantation. They reported various histological alterations as tubular atrophy, nodular arteriolar hyalinosis, tubular vacuolization, luminal narrowing, interstitial fibrosis, focal or global segmental sclerosis, and micro-calcifications in 79.2%–100% of cases [7], limiting the reward of minimal early acute rejection and short-term benefits of renal function. Moreover, CNIs have been kindred with burgeoning of various cardiovascular risk factors such as hyperlipidemia, hypertension, and new-onset diabetes mellitus following transplantation (NODAT) [8, 9].

Howbeit, the real associated confront with immunosuppression therapy is to perpetuate the balance of immunosuppression need in order to turn aside any rejection episode whilst keeping the toxicities at minimum. The neoteric immunosuppressive agents such as the mammalian target of rapamycin (mTOR) inhibitors, sirolimus (SRL), and everolimus (EVR), act similar to CNIs. These are formulated in present immunosuppressive regimen because of their less non-nephrotoxic profile [10, 11].

Calcineurin inhibitors as Tac and CsA couple with the intracellular proteins called FKBP and immunophilins to form complex that shuts off the corollary of calcineurin-mediated pathway. Calcineurin normally potentiates immunological pathway, *i.e.*, intracellular processes associated with the activation of T-lymphocytes. The inhibition of this sequence minimizes the production of interleukin-2 and impedes the proliferation of T-cells [12, 13]. 

By the same token, mTOR inhibitors like SRL and EVR also form a complex with FKBP to take the edge off from the T-cell activation by blocking growth-factor-mediated cell proliferation in the reaction to an alloantigen [14-17]. The unambiguous immunological characteristics and finite nephrotoxic potential of mTOR inhibitors have triumphed the use of CNIs in renal transplantation [18-21].

The main objective of this review was to cynosure the medium-term, *i.e.*, one-year benefit of early conversion to mTOR inhibitors with or without CNI in renal transplant recipients in terms of graft function and survival.

## MATERIALS AND METHODS

We performed the present systemic review following registration in PROSPERO, an international database of prospectively registered systematic reviews (CRD 42017054458). An extensive search of all the published literature on the role of early conversion to mTOR inhibitors as an alternative to CNI has been made on PubMed, EMBASE, Cochrane, Crossref, and Scopus on August 30, 2016. The search covered the period from January 1, 2001 (the year of the first reported early CsA withdrawal with SRL in the literature) to September 30, 2016 [22]. The following medical subject headings (MeSH) terms were used in our search: “adverse events,” “calcineurin inhibitors,” “cyclosporin,” “everolimus,” “graft rejection,” “graft survival,” “kidney transplantation,” “mTOR inhibitors,” “sirolimus,” “tacrolimus” were searched.

Inclusion Criteria

The original English literature articles published between January 1, 2001 and September 30, 2016 were included. Only studies that systematically and quantitatively assessed the graft function and graft survival of ≥12 months following early conversion to mTOR inhibitors with or without CNI in different randomized clinical trials were analyzed. All kind of comparative studies, retrospective and prospective were included. We excluded editorials, reviews, and letters (Table 1). 

**Table 1:** Criteria for the inclusion of early mTOR inhibitor conversion studies

Study design	Prospective cohort design with a well-defined study population
Study group	Post-renal transplantation
Conversion time	Period of 2 weeks to 6 months post-transplantation
Study size	>30 patients
Length of follow-up	Any
Source	Peer-reviewed journals
Language	English
Outcome measure	Patient safety, exposure-response relationships, adverse events, and graft function and long-term survival

Data Extraction

**Figure 1:**
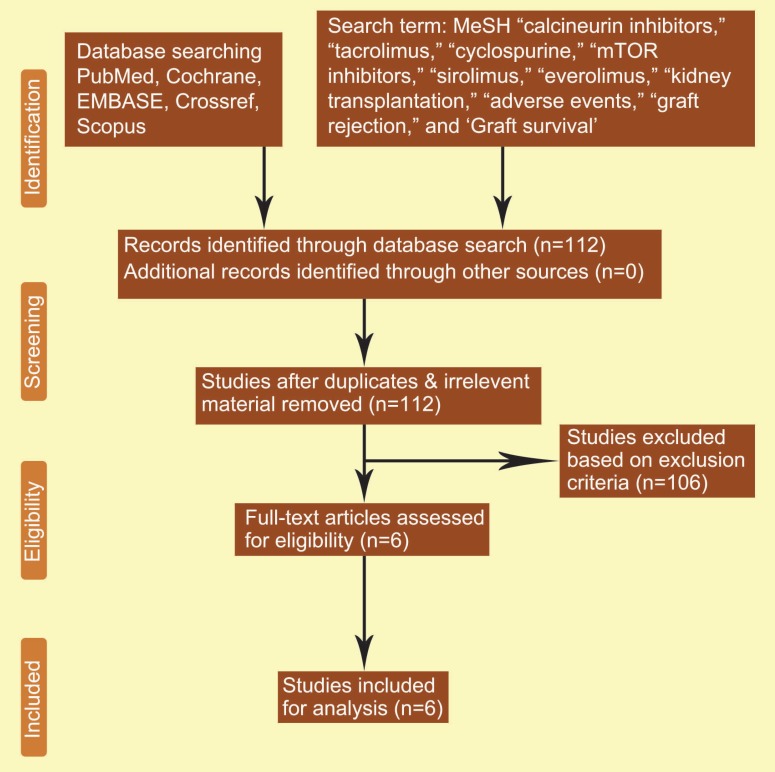
Search strategy and study selection used in this systematic review as per PRISMA protocol

Two separate physicians, KJ, and IR, reviewed all the retrieved articles. Disagreements were resolved through discussion; when the disagreement could not be resolved by discussion, the issue was examined by the third author (TK). We analyzed all papers with empirical studies using a standardized quality assessment tool and pre-specified inclusion and exclusion criteria. The present meta-analysis was performed using the Preferred Reporting Items for Systematic Reviews and Meta-analyses (PRISMA) guidelines and registered in PROSPERO (Fig 1).

Statistical Analysis

The quality assessment of diagnostic accuracy studies-II (QUADAS-II) based analysis was done to assess the internal validity of the pre-specified inclusion and exclusion criteria of the various studies. QUADAS-II is an evidence-based bias assessment tool to evaluate the quality of diagnostic accuracy studies in a systematic review.

A total of six peer-reviewed multi-institutional studies were included in the present meta-analysis. We reviewed each study comprehensively and data were extracted for the outcomes such as patient safety, exposure-response relationships, adverse events, and various shortcomings or weaknesses to improve the graft functioning and long-term survival (Table 2).

**Table 2:** Summary of various parameters in different early conversion clinical trials

Table 2: Summary of various parameters in different early conversion clinical trials					
Authors	Study design		Time of conversion	Group 1	Group 2
A) Everolimus					
Budde, *et al*, 2011, (ZEUS Study) [23]	Multicenter randomized trial (n=300), 12 months, 36 months, 5 years	4.5^th^ month		EVR (C0, 6–10 ng/mL) Induction: Basiliximab (n=155)	CsA ( C0, 120–180 ng/mL till 4.5–6 months then decreased to 100–150 ng/mL) Induction: Basiliximab (n=145)
Mjornstedt, *et al*, 2012, (CENTRAL trial) [24]	Multicenter randomized trial (n=269), 5 years	7^th^ week		EVR (C0, 6–10 ng/mL) + MMF (1.4 g/d till 2 weeks then decreased to 1.08 g/d) + S (n=92)	Low CsA ( C0, 75–200 ng/mL till 2 weeks then decreased to 50–150 ng/mL) + MMF (1.4 g/d) + S (n=90)
B) Sirolimus					
Lebranchu, *et al*, 2009, (CONCEPT Study) [25]	Multicenter randomized trial (n=193), 12 months, 48 months	3^rd^ month		SRL (C0, 8–15 ng/mL till 39 weeks then decreased to 5–10 ng/ mL) + MMF + S Induction: Daclizumab (n=95)	CsA (C0, 500–800 ng/mL) + MMF + S Induction: Daclizumab (n=97)
Guba, *et al*, 2010, (SMART Trial) [26]	Multicenter randomized trial (n=140), 12 and 36 months	10–24^th^ day		SRL (C0, 8–12 ng/mL then decreased to 5–10 ng/ mL) + MMF (1.5 g/d) + S Induction: ATG (n=69)	CsA (C0, 150–200 ng/mL then decreased to 100–150 ng/ mL) + MMF (2 g/d) + S Induction: ATG (n=71)
Weir, *et al*, 2010, (Spare the Nephron Trial) [27]	Multicenter randomized trial (n=299), 2 years	Within 115 days		MMF + SRL (n=148)	MMF + CNI (n=151)
Heilman, *et al*, 2011 [28]	Multicenter randomized trial (n=122), 24 months	1 month		SRL (C0, 9.8±3.6 ng/mL) + MMF + S Induction: Basiliximab (n=62)	TAC (C0, 6.9±4.6 ng/mL) + MMF + S Induction: Basiliximab (n=60)

## RESULTS

The initial search retrieved 112 articles of interest. However, following diligent assessment, we excluded 98 articles. Eventually, only six articles matched the previously laid inclusion criteria, *i.e*., ZEUS trial (2011), CENTRAL trial (2012), CONCEPT trial (2009), SMART trial (2010), Spare the Nephron trial (2010), and Heilman, *et al* (2011) (Table 2) [23-28]. Comprehensive data of these studies with focus on renal function, BPAR, survival and adverse events, are presented in Table 3.

**Table 3:** Summary of 12 months outcomes in different early conversion clinical trials

Authors	Renal function (Gp 1 *vs*. Gp 2)	BPAR (Gp 1 vs. Gp 2)	Adverse event (Gp 1 *vs*. Gp 2)	Remarks
A) Everolimus				
Budde, *et al*, 2011, (ZEUS Study) [23]	12 Months Sr. Cr: 141.7±44 *vs*. 137.0±43 µmol/L (p=NS) eGFR: 71.8±18 *vs*. 61.2±16 mL/min (p<0.001)	9.7% *vs*. 3.4% (p=0.03)	SAE/Infection: 61% *vs*. 59% (p=NS) UTI: 57.0% *vs*. 53% (p=NS) Diarrhea: 36% *vs*. 27% (p=NS) HPL: 14% *vs*. 10% (p=NS)	Graft survival: 100% *vs*. 100% (p=NS) Patient survival 100% *vs*. 99% (p=NS)
Mjornstedt, *et al*, 2012, (CENTRAL trial) [24]	12 Months Sr. Cr: 122.0±35 *vs*. 132.0±45 µmol/L (p=NS) eGFR: 68.1±21.5 *vs*. 69.4±22.9 mL/min (p=NS)	27.5% *vs*. 11.0% (p=0.004)	SAE/Infection: 53.9% *vs*. 38.0% (p=0.025) CMV infection: 8.8% *vs*. 13.0% (p=NS) Edema: 29.4% *vs*. 21.0% (p=NS) Anemia: 16.7% *vs*. 6.0% (p=0.02) HPL: 12.7% *vs*. 9.0% (p=NS) Proteinuria: 4.9% *vs*. 0 % (p=0.06) Acne: 12.7% *vs*. 2.0 % (p=0.006) Mouth ulceration: 12.7% *vs*. 2.0% (p=0.001)	Graft survival: 100% *vs*. 100% (p=NS) Patient survival 98% *vs*. 98% (p=NS)
B) Sirolimus				
Lebranchu, *et al*, 2009, (CONCEPT Study) [25]	12 Months: Sr. Cr: 117.4 *vs*. 132.3 µmol/L (p<0.001) eGFR: 68.9 *vs*. 64.4 mL/min (p=0.017)	16.8% *vs*. 8.2% (p=NS)	Peripheral edema: 28.1% *vs*. 22.6% (p=NS) SAE/Infection: 60% *vs*. 44% (p=0.025) Diarrhea: 30.2% *vs*. 9.2% (p<0.001) Dyslipidemia: 5.20% *vs*. 4.12% (p=NS) Proteinuria: 9.3% *vs*. 3.09% (p=NS) NODAT: 3.1% *vs*. 2.06% (p=NS) Aphthous stomatitis: 45.8% *vs*. 5.15% (p<0.001)	Graft survival: 99% (p=NS) Patient survival 97% (p=NS)
Guba, *et al*, 2010, (SMART Trial) [26]	12 Months: Sr Cr: 111.5 ± 45 mg/dl vs. 142.6 ± 74 mg/dl (p=0.004) eGFR: 64.5 ± 25.2 vs. 53.4 ± 18.0 ml/min (p=0.001).	17.4% *vs*. 15.5% (p=NS)	Wound healing disorder: 10.1% *vs*. 11.3% (p=NS) Infection: 52.2% *vs*. 60.6% (p=NS) CMV: 7.3% *vs*. 28.2% (p<0.001) HPL: 20.3% *vs*. 7.0% (p=0.02) Diarrhea: 13.0% *vs*. 9.9% (p=NS) Lymphocele: 27.5% *vs*. 23.9% (p=NS)	Graft survival: 99% *vs*. 97% (p=NS) Patient survival 99% *vs*. 99% (p=NS)
Weir, *et al*, 2010, (Spare the Nephron Trial) [27]	12 Months Sr. Cr: 126.2 ± 82.8 vs. 145.0 ± 96.5 µmol/L (p=NS) eGFR: 74.6 ± 17.9 vs. 71.5 ± 21.2 ml/min (p=0.06)	7.4% *vs*. 6.0% (p=NS)	Infection: 16.2% *vs*. 18.3% (p=NS) HPL: 24.3% *vs*. 10.5% (p<0.001) CMV: 4.7% *vs*. 9.2% (p=NS) Polyoma virus: 2% *vs*. 4% (p=NS) Diarrhea: 29.7% *vs*. 9.8% (p=0.001) Malignancy: 4.7% *vs*. 6.5% (p=NS)	Graft survival: 98% *vs*. 97.4% (p=NS) Patient survival 100% *vs*. 98% (p=NS)
Heilman, *et al*, 2011 [28]	12 Months Sr. Cr: 96.1 ± 28 vs. 106.1 ± 61 µmol/L (p=NS) eGFR: 63.0 ± 19.1 vs. 59.8 ± 18.9 ml/min (p=NS)	13% *vs*. 5% (p=NS)	CMV: 13% *vs*. 13% (p=NS) Polyoma virus: 2% *vs*. 4% (p=NS)	NA

## DISCUSSION

The inception of mTOR inhibitors in early post-transplantation period should be considered when the immunological risk is minimum and that of CNI-related toxicity has not established [29, 30]. Based on these facts, several CNI-free or minimized dosing regimens have been tried to limit the nephrotoxic adverse effects. The menace of heightened rejection risk with *de novo* use of CNI-free protocols has been mitigated with the early introduction of mTOR inhibitors. However, the evidence towards optimal time of conversion to mTOR inhibitor-based immunosuppression is not clear. At the same time, the present literature takes up the cudgels for early conversion to mTOR inhibitors within the six months of transplantation, whereas inducement of conversion following month six is not that beneficial. The major encumbrance in the anticipated outcome following late conversion might be due to the already established CNI-related nephrotoxicity [23, 25].

Considering the present evidence, mTOR inhibitors should be commenced within a period of two weeks to six months, *i.e.*, after the period of increased risk for rejection and wound infection has been ended.

A multicenter randomized trial (ZEUS study) conducted by Budde, *et al*, reported early conversion to EVR from CsA 4.5 months after renal transplantation. They randomized 269 patients into two groups. The first group received EVR with MMF, while another group was maintained on gradually tapered lower doses of CsA with MMF. They demonstrated significant improvement in GFR at 12 months following change to EVR (71.8±18 *vs*. 61.2±16 mL/min; p<0.001); BPAR was higher in the EVR group (13.9% *vs.* 7.5%; p=0.09). However, they heralded no difference in terms of graft and patient survival [23].

In 2012, Mjornstedt, *et al*, did a CENTRAL trial to study the effect of early conversion from CsA to EVR seven weeks post-transplantation. Two-hundred and two patients who were randomized into EVR group (C_0_, 3–8 ng/mL) and CsA (C_0_, 75–200 ng/mL for two weeks then reduced, further maintained at 50–150 ng/mL) with oral steroids and MMF group. They reported lower serum creatinine in mTOR inhibitor group (122.0±35 *vs*. 132.0±45 µmol/L, p>0.05) although there was no significant change in GFR in EVR group compared to CsA group (68.1±21.5 *vs*. 69.4±22.9 mL/min, p>0.05) at 12 months. At the same time, the reported incidence of BPAR was significantly higher in EVR group compared to CsA group (27.5% *vs.* 11.0%, p=0.004); the survival outcomes were similar at 12 months. The reported adverse effects as proteinuria, anemia, hyperlipidemia, acne, and mouth ulceration were significantly more common in the EVR group [24].

Lebranchu, *et al*, conducted the CONCEPT study to explore the effects of introduction of SRL instead of CsA in the 3^rd^ month post-transplantation. The found significant improvement in eGFR (68.9 *vs.* 64.4 mL/min) and significant decrease in serum creatinine level (117.4 *vs.* 132.3 µmol/L, p<0.001) in the SRL group at 12 months. They reported similar BPAR for the entire period of observation. The reported adverse effects such as diarrhea, SAE, aphthous stomatitis, proteinuria, and new-onset diabetes mellitus were either significantly higher or more in the SRL group [25].

Guba, *et al*, completed the multicenter randomized SMART trial, by introducing very early conversion to SRL only 10 to 24 days from CsA following the renal transplantation. A total of 141 patients were randomized into two groups SRL with MMF and steroid, while the second group was maintained on gradually tapered lower doses of CsA with MMF and steroid. They demonstrated statistically significant improvement in renal function, eGFR (64.5±25.2 *vs*. 53.4±18 mL/min; p=0.001) with significant reduction in serum creatinine (111.5±45 *vs*. 142.6±74 µmol/L; p=0.004) for the SRL group at 12 months. Although the reported incidence of BPAR (17.4% *vs*. 15.5%, p>0.05) was similar in both groups; the graft and patient survival rates were quite similar. Furthermore, the recipients in the SRL group reported a significantly higher number of adverse effects such as acne, hyperlipidemia, and lower number CMV viremia whereas the incidence of BPAR was similar in both groups (20.2% *vs.* 19.7%; p>0.05) [26]. 

In 2010, Weir, *et al*, managed Spare the Nephron Trial, where 299 kidney transplant recipients were randomized into two groups following 115 days of renal transplantation. The first group received SRL with MMF while the second group was maintained on CNI and MMF. They noticed significant improvement in renal function with regard to higher eGFR (74.6±17.9 *vs*. 71.5±21.2 mL/min; p=0.06) and lower serum creatinine (126.2±82.8 *vs*. 145.0±96.5 µmol/L, p>0.05) in the SRL group. The reported survival in terms of the patient and graft were comparable in both groups. However, patients in the SRL group reported a significantly higher number of adverse effects as hyperlipidemia and diarrhea [27].

In the 2011 study by Heilman, *et al*, SRL was instituted in the first month of the post-transplantation period. They reported significant improvement in eGFR (63.0±19.1 *vs.* 59.8±18.9 mL/min; p>0.05) with lower serum creatinine in the SRL group at 12 months whilst the reported BPAR was similar in both groups [28]. 

As a general rule, early CNI abolition by mTOR inhibitor-based regimen gives the impression of being a more pragmatic and productive approach towards immunosuppressive treatment of renal transplant recipients. Nevertheless, taking into account the high rejection rate encountered in these studies, it will be prudent not to put forward this regimen to patients with moderate to high immunological risk. Nonetheless, additional studies with long duration of follow-up are much warranted to confirm this judgment [31-33].

## CONCLUSIONS

Albeit the literature on the Tac minimization strategies are inadequate, the current attestation recommended early introduction of mTOR inhibitors and substantial CNI minimization and found them better in terms of renal function. Howbeit, this stratagem has been subservient to other regimens as MMF/Tac regarding BPAR and patient/graft survival and it is inferior to MMF/Tac and SRL/MMF regimens in terms of renal function. Therefore, it is not sagacious approach to extend mTOR inhibitors to patients with moderate to high immunological risk. 

## CONFLICTS OF INTEREST:

None declared. 
